# Receiver operating characteristic (ROC) to determine cut-off points of clinical and biomolecular markers to discriminate mortality in severe COVID-19 living at high altitude

**DOI:** 10.1186/s12890-023-02691-2

**Published:** 2023-10-18

**Authors:** Jorge Luis Vélez-Páez, Lucy Baldeón-Rojas, Cristina Cañadas Herrera, Mario Patricio Montalvo, Fernando Esteban Jara, Santiago Aguayo-Moscoso, Wendy Tercero-Martínez, Lenin Saltos, Glenda Jiménez-Alulima, Verónica Guerrero, Jorge Pérez-Galarza

**Affiliations:** 1 Pablo Arturo Suarez Hospital, Intensive Care Unit, Clinical Research Center, Quito, Ecuador; 2 Faculty of Medical Sciences, Central University of Ecuador, Quito, Ecuador; 3 Research Institute of Biomedicine, Central University of Ecuador, Quito, Ecuador

**Keywords:** ROC curve, Cut-off, Odds ratio, Severe COVID-19, Inflammatory markers, High altitude

## Abstract

**Background:**

In 2020, Ecuador had one of the highest death rates because of COVID-19. The role of clinical and biomolecular markers in COVID disease prognosis, is still not well supported by available data. In order for these markers to have practical application in clinical decision-making regarding patient treatment and prognosis, it is necessary to know an optimal cut-off point, taking into consideration ethnic differences and geographic conditions.

**Aim:**

To determine the value of clinical and biomolecular markers, to predict mortality of patients with severe COVID-19 living at high altitude.

**Methods:**

In this study, receiver operating characteristic (ROC) curves, area under the curve (AUC) of ROC, sensitivity, specificity and likelihood ratios were calculated to determine levels of clinical and biomolecular markers that best differentiate survivors versus non-survivors in severe COVID subjects that live at a high altitude setting.

**Results:**

Selected cut-off values for ferritin (≥ 1225 ng/dl, *p* = 0.026), IL-6 (≥ 11 pg/ml, *p* = 0.005) and NLR (≥ 22, *p* = 0.008) at 24 h, as well as PaFiO2 (≤ 164 mmHg, *p* = 0.015), NLR (≥ 16, *p* = *p* = 0.013) and SOFA (≥ 6, *p* = 0.031) at 72 h, appear to have good discriminating power to differentiate survivors versus non-survivors. Additionally, odds ratios for ferritin (OR = 3.38); IL-6 (OR = 17.07); PaFiO2 (OR = 4.61); NLR 24 h (OR = 4.95); NLR 72 h (OR = 4.46), and SOFA (OR = 3.77) indicate increased risk of mortality when cut-off points were taken into consideration.

**Conclusions:**

We proposed a straightforward and understandable method to identify dichotomized levels of clinical and biomolecular markers that can discriminate between survivors and non-survivors patients with severe COVID-19 living at high altitudes.

**Supplementary Information:**

The online version contains supplementary material available at 10.1186/s12890-023-02691-2.

## Background

Death rates caused by COVID-19 pandemic were considerably high in Latin America, a region with notable levels of socioeconomic inequality [1]. Because health systems lacked diagnostic tools and resources, it was challenging to control disease outbreaks at community level, as result, in 2020 there was a high rate of hospital admissions [2]. In Ecuador, most COVID-19 patients (around 80%) had mild to moderate symptoms; however, the remaining 20% developed a severe condition that needed intensive care unit (ICU) which dramatically raises the risk of mortality [3, 4].

Numerous studies have identified demographic, clinical and molecular risk factors that contribute to the severity of COVID-19 disease, including older age, underlying health condition (hypertension, obesity, and/or diabetes), low serum albumin, high neutrophil-to-lymphocyte ratio (NLR), IL-6, LDH and C-reactive protein, [5, 6]. Analysis of these indicators is crucial for early sickness diagnosis, development of target treatments, and disease prognosis.

Thus, clinical and biomolecular markers have prognostic significance; however, have been demonstrated to differ dramatically across COVID patients. A particularly severe immune reaction known as the "cytokine storm", which is characterized by the production of increased levels of inflammation and causes systemic disease can occur in some COVID-19 patients. Additionally, ecological research suggested that geographical factors may have an impact on survival rate, suggesting that high altitude living may be associated with lower COVID-19 morbidity and mortality [7]. Nevertheless, it is not yet clear, how altitude would protect against COVID-19 mortality and how this might apply in a clinical context [8–11].

Considering that the existing data still does not provide strong evidence for the value of clinical and biomolecular indicators in COVID disease prediction; it is essential to identify the ideal cut-off point, in order for clinical decision-making regarding patient treatment and prognosis to be feasible as well as to anticipate categorization of severe COVID patients into those with low vs. high risk of death.

## Materials and methods

The study was conducted in Quito, Ecuador's capital city, which is located at an altitude of 2,850 m above sea level, in an intensive care unit (ICU) at a secondary hospital that exclusively treats COVID-19 patients.

### Study design

A retrospective cohort study was carried out from April 1, 2020, to March 1, 2021, using secondary anonymized data from a clinical database of adults with confirmed COVID-19, identified by a positive real-time reverse transcription polymerase chain reaction (RT-PCR), admitted to the intensive care unit (ICU).

### Population and sample size

**Fig. 1:**
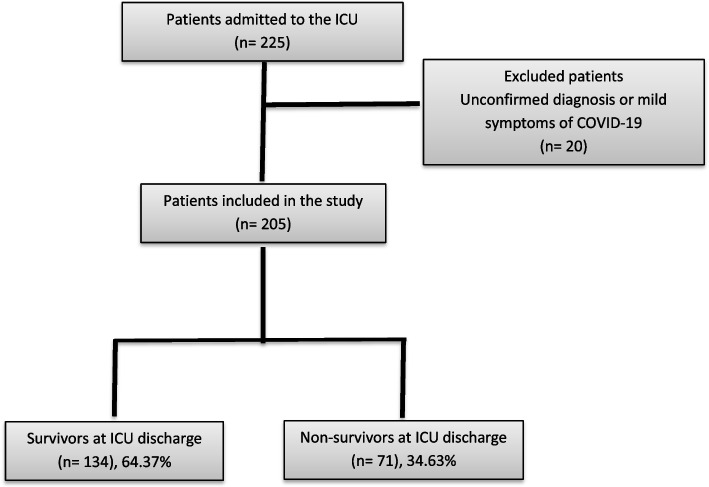
Population and Sample size flow chart of the study

In this study, patients with COVID-19 diagnosis who were admitted to the ICU were taken into account. No formal sample size calculation was performed due to the exploratory, descriptive, and retrospective nature of the study. During the study, 225 were admitted to the ICU. Nevertheless, only 205 participants met the inclusion criteria; 20 individuals were left out of the study because lack of molecular diagnosis or non-severe symptoms (Fig. 1).

### Inclusion criteria

Adult patients older than 18 years old, hospitalized at ICU who underwent invasive mechanical ventilation with diagnosis of severe COVID-19, determined by molecular test (RT-PCR) or antigen test and a CO-RADS score of 4 or 5 (indicating high probability of COVID-19) were included.

### Exclusion criteria

Patients with solid organ or hematological malignancies, individuals with mild to moderate clinical symptoms of COVID-19, or with respiratory symptoms not related to SARS-CoV-2 infection were excluded from the study.

### Data collection

We documented demographic characteristics such as age, sex and comorbidities (diabetes mellitus, arterial hypertension and obesity). The rest of clinical, biomolecular and, ventilatory markers were documented at 24, 48 and 72 h. Clinical scales such as the Sequential Organ Failure Assessment (SOFA) and the Acute Physiology and Chronic Health Evaluation II (APACHE II) were gathered from clinical records. Mechanical ventilation data was measured in a quasi-static flow environment, with the patients sedated and muscle relaxants provided. In Mindray SynoVent E5 and Hamilton C3 ventilators, they were set in a mandatory volume-controlled sequence with a tidal volume of 8 ml/kg of ideal weight and a respiratory rate of 15 breaths per minute.

Biomolecular markers including D-dimer (NV: 0.0–500 ng/ml), ferritin (NV: 22–322 ng/ml), LDH (NV: 135–214 U/L), and IL-6 (NV: 0.0–3.4 pg/ml) were recorded. Ferritin and IL-6 were evaluated using chemiluminescence in blood samples collected in tubes without anticoagulant (Inmulite 2000 XPi, USA). Photometry (Advia 1800) was used to assess the levels of LDH, and a fluorescence enzyme-linked immunosorbent assay (ELISA) was used to measure D-dimer in blood collected in a sodium citrate tube (Cobras Pro, Module 503). An automated hematology analyzer was used to perform a routine hemogram on blood taken in K3 tubes with EDTA anticoagulant (Advia 2120i, USA). Lymphocyte, platelet, neutrophil and eosinophil counts, as well as the neutrophil-to-lymphocyte ratio (NLR), were derived from hemogram. The survival or non-survival status was documented as well. Data was collected at ICU admission, after 24, 48, and 72 h*.*

### Statistical analysis

Continuous variables were reported as mean with standard deviation (SD), while categorical variables were presented as frequencies. The normal distribution of continuous variables was evaluated using the Kolmogorov–Smirnov tests. For quantitative variables, the Student's t-test for independent samples or the Mann–Whitney test for the comparison between survivor and non-survivor groups was used as appropriate. The estimation of any association between laboratory variables and survivors versus non-survivors was assessed with a preliminary univariate analysis (chi square test with Yates correction or Fisher’s exact test). An odds ratio greater than one was used to indicate that the outcome was more likely to occur in one group. We used ROC curve analysis to predict mortality, determining cut-off points using the Younden index for the measured variables, complemented with the calculated area under the ROC curve (AUC) as a quantitative measure of the discrimination power of markers between two groups. At the multivariate level, the Wald method of regression of the forward logistic procedure was used, to determine the predictors of mortality at ICU discharge in patients with severe COVID-19 admitted to the ICU using the variables that were statistically associated in the bivariate analyses. Statistical significance was established for a value of *p* < 0.05. All statistical analyzes were performed using the R version 4.1.2 software.

## Results

A total of 205 COVID-19 individuals were studied. Table 1 shows demographic and clinical features between non-survival and survival groups (34,6% vs. 65,4%). The mean age was 51.7 years old, showing significant differences between non-survivors and survivors (58.07 vs. 48.37 years; *p* = 0.000). Male were more affected but there was not gender significant differences in survival.

**Table 1:** Clinical characteristics in non-survival and survival groups

Characteristics	Total	Condition at discharge		*p*-value
		**No-survival**	**Survival**	
Age (mean (SD)) years^1/^	51,73 (12,40)	58,07 (11,02)	48,37 (11,8)	0,000*
**Gender (n (%))**^**2/**^				
Male	146 (71,22)	56 (38,36)	90 (61,64)	0,078
Female	59 (28,78)	15 (25,42)	44 (74,58)	
**T2DM (%))**^**2/**^				
Yes	28 (13,66)	11 (39,29)	17 (60,71)	0,578
No	177 (86,34)	60 (33,9)	117 (66,1)	
**HBP (n (%))**^**2/**^				
Yes	34 (16,59)	15 (44,12)	19 (55,88)	0,203
No	171 (83,41)	56 (32,75)	115 (67,25)	
**Obesity (n (%))**^**2/**^				
Yes	54 (26,34)	15 (27,78)	39 (72,22)	0,217
No	151 (73,66)	56 (37,09)	95 (62,91)	
BMI (mean (SD))^3/^	29,66 (4,54)	29,04 (4,78)	30,01 (4,38)	0,060
APACHE II at admission (mean (SD))^3/^	17,24 (6,04)	18,79 (6,47)	16,42 (5,66)	0,010*
**SOFA (mean (SD))**^**3/**^				
24 h	7,55 (2,93)	8,45 (3,33)	7,07 (2,57)	0,006*
48 h	5,93 (2,65)	6,83 (2,87)	5,48 (2,42)	0,002*
72 h	5,18 (2,71)	6,47 (2,9)	4,56 (2,39)	0,000*
Corticosteroids use (n (%))^2/^	183 (89,27)	63 (34,43)	120 (65,57)	0,857
Heparin use (n (%))^2/^	161 (78,92)	59 (36,65)	102 (63,35)	0,175
Hospitalization days (mean (SD))^3/^	10,27 (7,33)	10,55 (7,34)	10,13 (7,35)	0,556

*T2DM* Type 2 diabetes mellitus, *HBP* High blood pressure, *BMI* Body mass index, *APACHE* Acute Physiology and Chronic Health Evaluation II Score, *SOFA* Sequential Organ Failure Assessment Score, *SD* Standard Deviation; *significant differences (*p*-value < 0.05). ^1/^ T-test ^3/^ Mann Whitney U test; ^2/^ comparison with no-survival condition based on Chi-square test or exact Fisher test; *OR* Odds Ratio, *CI* Confidence interval

In all groups, obesity was the most common comorbidity (26.34%), followed by hypertension (16.59%) and diabetes (13.66%). APACHE II at admission shows significant differences between non-survivors and survivors (18.76 vs. 16.42; *p* = 0.010). SOFA demonstrated significant differences between non-survivors and survivors at 24, 48, and 72 h (8.45 vs. 7.07; *p* = 0.006), (6.83 vs. 5.48; *p* = 0.002), and (6.47 vs. 4.56; *p* = 0.000) respectively. The average length of hospital stay was 10.27 days, with no significant differences between non-survivors and survivors (Table 1).

Regarding mechanical ventilation parameters, the most frequent admission ventilation mode was pressure-controlled (92.20%). Prone ventilation was used in 61.46% patients and muscle relaxant administration in 63.37%. We show significant differences between non-survivors and survivors at 24 h for PCO2, at 48 h for PaFiO2, PEEP and PaFiO2, at 72 h for PCO2, PaFiO2, Plateau and Driving pressure. Number of days spent on mechanical ventilation (10.39 vs. 7.70; *p* = 0.002), and successful extubation (1, 74% vs. 98.26%; *p* = 0.000) were also significant between non-survivors and survivors (Supplementary Table 1). Furthermore, the cut-off points that predicted mortality in the ROC curve using the Youden index of mechanical ventilation parameters were positive for the following parameters: PEEP at 48 h (≥ 8.50 cmH2O, sensitivity 43% and specificity 75%). Plateau pressure at 72 h (≥ 19.50 cmH2O, sensitivity 39% and specificity 82%). Driving pressure at 72 h (≥ 13.50 cmH2O, sensitivity 56% and specificity 63%). Maximum PCO2 at 24 h (≥ 41.50 mmHg, sensitivity 61% and specificity 54%) and Maximum PCO2 at 72 h (≥ 35.50 mmHg, sensitivity 90% and specificity 31%) (Supplementary Fig. 1).

In regards to biochemical markers, we showed significant differences between non-survivors and survivors in ferritin at 24 h (1,233.61 ng/dl vs. 1,055.79 ng/dl, *p* = 0.049). LDH at 24 h, (963.30 U/L vs. 837.29 U/L, *p* = 0.012), and IL-6 (103.93 pg/ml vs. 42.59 pg/ml; *p* = 0.001). There were no significant differences in LDH (48 h), ferritin (48 and 72 h) and D-dimer (24, 48, and 72 h) (Table 2) (Supplementary Fig. 2).

**Table 2:** Biomarkers characteristic in non-survival and survival groups

Biomarker	Total	Condition at discharge		*p*-value
		**No-survival**	**Survival**	
D-dimer 24 h(mean (SD)) ng/ml^2/^	2651,37 (5111,96)	3449,75 (7995,3)	2224,76 (2397,36)	0,202
D-dimer 48 h(mean (SD)) ng/ml^2/^	2667,57 (2880,31)	3647,66 (3629,45)	2165,86 (2275,08)	0,063
D-dimer 72 h(mean (SD)) ng/ml^2/^	2649,47 (2340,94)	3392,09 (2913,78)	2252,25 (1889,5)	0,189
Ferritin 24 h (mean (SD)) ng/ml^2/^	1117,68 (489,78)	1233,61 (435,71)	1055,79 (507,1)	0,049*
Ferritin 48 h (mean (SD)) ng/ml^2/^	1109,64 (464,48)	1181,81 (400,23)	1076,92 (489,54)	0,323
Ferritin 72 h (mean (SD)) ng/ml^2/^	1236,65 (777,84)	1228,2 (462,99)	1241,4 (913,77)	0,642
LDH 24 h (mean (SD)) U/L^2/^	881,17 (360,74)	963,3 (410,25)	837,29 (324,51)	0,012*
LDH 48 h (mean (SD)) U/L^1/^	775,05 (342,39)	838,89 (474,2)	742 (245,54)	0,128
IL-6 (mean (SD)) pg/ml^2/^	63,7 (113,77)	103,93 (161,29)	42,59 (70,28)	0,001*

*LDH* Lactate dehydrogenase, *IL* Interleukin, *SD* Standard deviation; * significant differences between means, based on ^1/^ T-test y ^2/^ Mann Whitney U test

As to hematological parameters, we show significant differences when compared non-survivors and survivors in lymphocytes count at 24, 48 and 72 h (571.23 × 103/ml vs. 727.86 × 103/ml; *p* = 0.000); (564.25 × 103/ml vs. 678.51 × 103/ml; *p* = 0.000) and (487.77 × 103/ml vs. 769.29 × 103/ml; *p* = 0.000). Platelets count at 24, 48, and 72 h (307,298.51 × ml vs. 350,231.34 × ml; *p* = 0.029), (307,298.51 × ml vs. 360,746.27 × ml; *p* = 0.007) and (311,727.27 × ml vs. 367,947.76 × ml *p* = 0.005). Neutrophils count at 24 h (11,753.52 × ml vs. 9,487.71 × ml; *p* = 0.024) and neutrophil lymphocyte ratio (NLR) at 24, 48, and 72 h (28.86 vs. 16.67; *p* = 0.000), (26.41 vs. 17.73; *p* = 0.000) and (32.60 vs. 20.11; *p* = 0.000) (Table 3) (Supplementary Fig. 2).

**Table 3:** Hemogram characteristic in non-survival and survival groups

Biomarker	Total	Condition at discharge		*p*-value
		**No-survival**	**Survival**	
Lymphocytes 24 h (mean (SD))^2/^	673,61 (374,55)	571,23 (412,3)	727,86 (342,26)	0,000*
Lymphocytes 48 h (mean (SD))^2/^	640,43 (465,85)	564,25 (620,45)	678,51 (361,84)	0,000*
Lymphocytes 72 h (mean (SD))^2/^	677,34 (524,13)	487,77 (253,12)	769,29 (593,34)	0,000*
Platelets 24 h (mean (SD))^2/^	337,926,83 (121,822,39)	314,704,23 (121,476,56)	350,231,34 (120,648,8)	0,029*
Platelets 48 h (mean (SD))^2/^	342,930,35 (126,045,41)	307,298,51 (114,267,9)	360,746,27 (128,265,45)	0,007*
Platelets 72 h (mean (SD))^2/^	349,395 (124,949,19)	311,727,27 (118,862,11)	367,947,76 (124,113,3)	0,005*
Neutrophils 24 h (mean (SD)) ^2/^	10,272,45 (5962,01)	11,753,52 (8278,7)	9487,71 (4081,38)	0,024*
Neutrophils 48 h (mean (SD))^2/^	9400,43 (4205,02)	9872 (3949,17)	9157,51 (4325,43)	0,335
Neutrophils 72 h (mean (SD))^2/^	11,083,67 (14,321,56)	12,949,55 (21,723,65)	10,143,62 (8403,68)	0,333
Eosinophils 24 h (mean (SD))^2/^	23,01 (48,88)	20,27 (35,25)	24,46 (54,81)	0,949
Eosinophils 48 h (mean (SD))^2/^	28,57 (64,17)	23,26 (41,46)	31,3 (73,18)	0,346
Eosinophils 72 h (mean (SD))^2/^	31,88 (74,32)	29,49 (60,98)	33,08 (80,36)	0,716
NLR 24 h (mean (SD))^2/^	20,89 (20,46)	28,86 (28,15)	16,67 (13,15)	0,000*
NLR 48 h (mean (SD))^2/^	20,66 (19,06)	26,41 (22,34)	17,73 (16,49)	0,000*
NLR 72 h (mean (SD))^2/^	24,25 (39,02)	32,6 (58,6)	20,11 (23,25)	0,000*

*SD* Standard deviation, *NLR* Neutrophil-to-lymphocyte ratio; * significant differences of means, based on ^1/^ T-test y ^2/^ Mann Whitney U test

ROC curve was used to examine which mechanical ventilation, biochemical, and hematological markers that were significant in the bivariate analysis could be predictors of death.

**Fig. 2:**
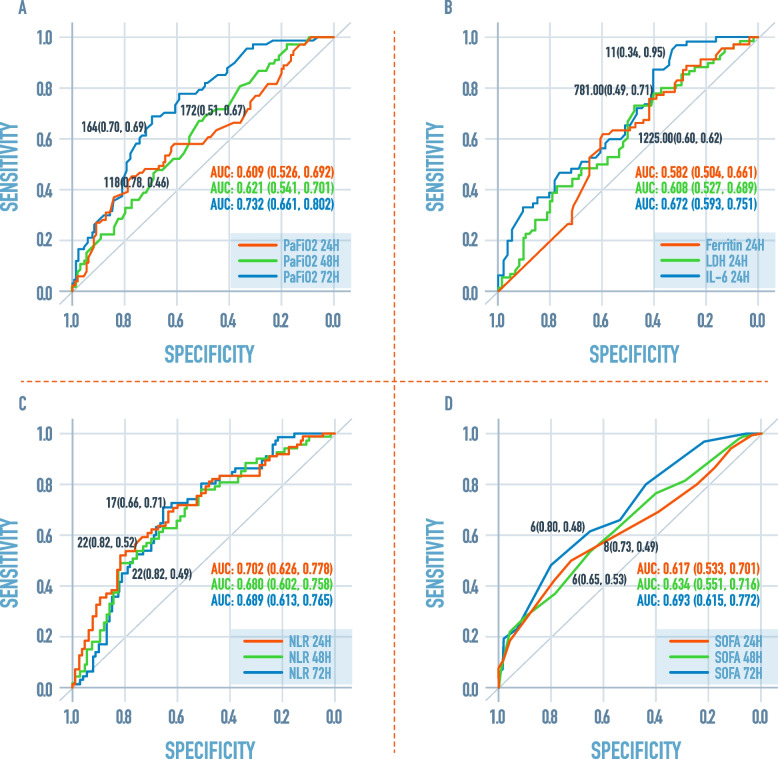
ROC curves showing the area under the curve (AUC) and cut-off points for the different variables associated with the non-survival condition in the bivariate analysis. **A** PaFiO2 at 24, 48 and 72 h; **B** Ferritin at 24 h, IL-6 at 24 h, and LDH at 24 h; **C** NLR 24, 48 and 72 h; **D** SOFA at 24, 48 and 72 h. PaFiO2: ratio of arterial oxygen partial pressure (PaO2 in mmHg) to fractional inspired oxygen; LDH: lactate dehydrogenase; IL: interleukin; NLR: neutrophil-to-lymphocyte ratio; SOFA: Sequential Organ Failure Assessment Score

ROC curve in Fig. 2A exhibits areas for PaFiO2 at 24, 48 and 72 h which were significant to predict mortality in patients with COVID-19 (0.609; 95% CI 0.526–0.692), (0.621; 95% CI 0.541–0.701), (0.732; 95% CI 0.661–0.802). The cut-off points that predicted mortality in the ROC curve using the Youden index for PaFiO2 at 24 h was ≤ 118 mmHg (sensitivity 46% and specificity 78%). At 48 h ≤ 172 mmHg (sensitivity 67% and specificity 51%) and at 72 h ≤ 164 mmHg (sensitivity 69% and specificity of 70%).

ROC curve in Fig. 2B presents ferritin at 24 h (0.582; 95% CI 0.504–6.661), IL-6 (0.672; 95% CI 0.593–0.851), and LDH (0.608; 95% CI 0.527–0.6893), which were significant in predicting COVID-19 mortality. The cut-off points that predicted mortality in the ROC curve using the Youden index for ferritin was ≥ 1225 ng/dl, (sensitivity 62% and specificity 60%), IL-6 ≥ 11 pg/ml (sensitivity 95% and specificity 34%) and LDH at 24 h ≥ 781 U/L (sensitivity 71% and specificity of 49%).

ROC curve in Fig. 2C indicates the areas for NLR at 24 h (0.702; 95% CI 0.626–0.778), at 48 h (0.680; 95% CI 0.602–0.758), and at 72 h (0.689; 95% CI 0.613- 0.765), which were significant in predicting COVID-19 mortality. The cut-off points that predicted mortality in the ROC curve using the Youden index for the NLR at 24 h was ≥ 22 (sensitivity 82% and specificity 52%), at 48 h ≥ 22 (sensitivity 82% and specificity 49%) and at 72 h ≥ 17 (sensitivity 71% and specificity 66%).

Besides, ROC curve in Fig. 2D shows the areas for SOFA at 24 h (0.617; 95% CI 0.533–0.701), at 48 h (0.634; 95% CI 0.551–0.716) and at 72 h (0.693; 95% CI 0.615- 0.7752), which were significant for predicting mortality. The cut-off points that predicted mortality in the ROC curve using the Youden index for SOFA at 24 h was ≥ 8 (sensitivity 73% and specificity 49%) and at 48 h ≥ 6, (sensitivity 53% and specificity 65%). Finally, the cut-off for NLR at 72 h was ≥ 6 (sensitivity 48% and specificity 80%).

To complete the analysis, univariate and multivariate logistic regression was used to determine the relationship between mortality from COVID-19 and the cut-off points for mechanical ventilation, biochemical and hemogram parameters. The results obtained showed that ferritin at 24 h ≥ 1225 ng/dl, IL-6 at 24 h ≥ 11 pg/ml, PaFiO2 at 72 h ≤ 164 mmHg, NLR at 24 h ≥ 22, and SOFA at 72 h ≥ 6 are the best predictors of COVID-19 mortality. In the multivariate analysis, the SOFA did not maintain its predictive level (*p* = 0,059). Therefore, taking into account the specified cut-off points, the probability that patients do not survive was for ferritin OR = 3.31 (CI 95% 1.40—7.79); IL-6 OR = 9.65 (CI 95% 1.94 – 47.13); PaFiO2 OR = 4.12 (CI 95% CI 1.74 – 9.76); for NLR 24 h OR = 7.29 ( CI 95% 3.04 – 17.49) (Table 4).

**Table 4:** Logistic regression to predict OR of mortality based on mechanical ventilation, biomolecular and hemogram markers

Variables	Univariate				Multivariate			
	**OR**	**CI-OR 95%**		***p*-value**	**OR**	**CI-OR 95%**		***p*-value**
		**Ll**	**Hl**			**Ll**	**Hl**	
**Significant variables**								
Ferritin 24 h ≥ 1225 ng/dl	2,46^a^	1,36	44,46	0,003*	3,31^a^	1,40	7,79	0,006*
IL-6 24 h ≥ 11 pg/ml	13,93^a^	3,23	60,06	< 0,001*	9,65^a^	1,94	47,13	0,006*
PaFiO2 72 h ≤ 164 mmHg	5,09^a^	2,69	9,62	< 0,001*	4,12^a^	1,74	9,76	0,001*
NLR 24 h ≥ 22	4,90^a^	2,57	9,36	< 0,001*	7,29^a^	3,04	17,49	< 0,001*
SOFA 72 h ≥ 6	2,96^a^	1,58	5,53	0,001*	2,29	0,97	5,41	0,059

*IL* Interleukin, *PaFiO2* ratio of arterial oxygen partial pressure (PaO2 in mmHg) to fractional inspired oxygen, *NLR* Neutrophil-to-lymphocyte ratio, *SOFA* Sequential Organ Failure Assessment Score; * significant variables *p*-value < 0,05, ^a^*OR* significant odds ratio, *CI* Confidence interval, *Ll* Lower limit, *Hl* Higher limit; based on logistic regression

**Fig. 3:**
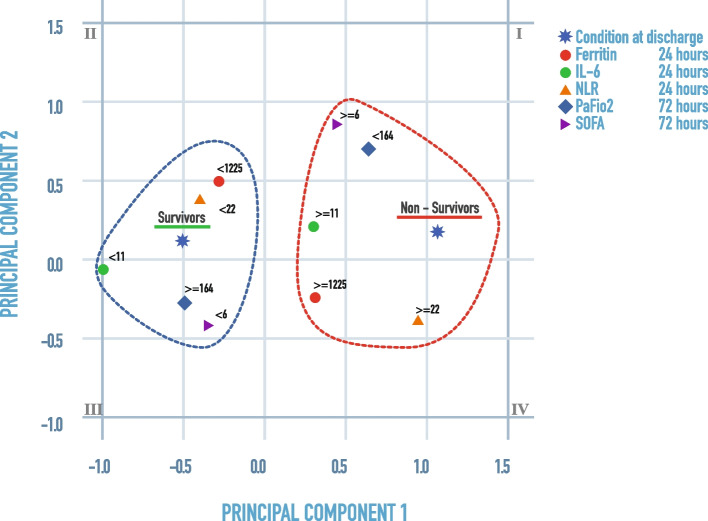
Multivariate relationship between discharge condition and prognostic parameters based on categorical principal component analysis (CATPCA). IL: interleukin; NLR: neutrophil-to-lymphocyte ratio; PaFiO2: ratio of arterial oxygen partial pressure (PaO2 in mmHg) to fractional inspired oxygen; SOFA: Sequential Organ Failure Assessment Score

Finally, categorical principal component analysis (CATPCA) was used to illustrate the associations discovered in the logistic regression into two-dimensional space. In Fig. 3, quadrants IV and I show the pattern of non-surviving patients (ferritin at 24 h > 1225 ng/dl, IL-6 at 24 h > 11 pg/mL, PaFiO2 at 72 h < 164 mmHg, NLR at 24 h > 22, and SOFA at 72 h > 6). On the other hand, quadrants II and III show the pattern of surviving patients (Ferritin at 24 h < 1225 ng/dl, IL-6 at 24 h < 11 pg/mL, PaFiO2 at 72 h > 164 mmHg, NLR at 24 h < 22and SOFA at 72 h < 6) (Fig. 3).

## Discussion

Our study presents a series of critically ill COVID-19 patients, who required intensive care unit, therefore mechanical ventilation. Male/female sex ratio was 2.5 to 1, non-survivors were older than survivors; and obesity, type 2 diabetes and high blood pressure were the most common comorbidities. These results are in agreement with those of earlier worldwide research that have identified similar demographic and metabolic comorbidities as important mortality risk factors [12–17]. Additionally, we show that, when compared to other Latin American nations, where the mortality rate was approximately 41.6%, our research group's death rate was relatively low (34%). This finding is in line with previous studies showing increase survival rates in critically sick patients when they reside at high altitudes [7, 18]. This observation might be explained by genetic and physiological adaptations based on by long-term hypoxic exposure. In our study, one out of every four hospitalized patients were obese, suggesting that the "obesity paradox" may provide some protection for patients who reside in high-altitude Quito, Ecuador (2,850 masl). This theory states that hypobaric hypoxia in obese people may provide some resistance to respiratory distress syndrome (ARDS) which is a hypoxemic illnesses often present in severe COVID-19 patients [7, 18–27].

ROC, sensitivity, specificity, as well as likelihood ratios were calculated to determine levels of clinical and biomolecular markers that best can differentiate survivors versus non-survivors in our group of severe COVID patients. The selected cut-off values for ferritin (≥ 1225 ng/dl, *p* = 0.026), IL-6 (≥ 11 pg/ml, *p* = 0.005) and NLR (≥ 22, *p* = 0.008) at 24 h, as well as PaFiO2 (≤ 164 mmHg, *p* = 0.015), NLR (≥ 16, *p* = *p* = 0.013) and SOFA (≥ 6, *p* = 0.031) at 72 h, appear to have the adequate discriminating power.

When individually analyzed IL-6 and NLR at 24 h showed the best sensitive rates (95% and 82% respectively). Additionally, PaFiO2 at 24 h and NLR and at 72 showed the best specificity rates (78% and 80% respectively).

Evidence suggests that IL-6 plays a central role in the cytokine storm driving immunological dysregulation. Similar to other studies, we described this inflammatory marker an important predictor of mortality. Available evidence has indicated elevated levels of IL-6 related with poor prognosis of COVID-19 patients [28, 29].However, Liu et al. reported that solely monitoring blood levels of IL-6 at early stages of COVID-19, may accurately predict disease severity but not mortality [30]. Interleukin IL-6 also regulates transferrin receptors as well as ferritin expression [31]. Iron deficiency may impair oxygen absorption and transport, exacerbating ARDS [32].

In the same line, NLR, an inflammatory marker, has been used for a very long time to predict morbidity and mortality in patients with conditions including sepsis, heart disease, and cancer [33]. In COVID-19 patients, it is well described that NLR also, can effectively predict mortality with high sensitivity (88.7%) and a specificity (95.4%) [34, 35], agreeing with our findings.

Our severe COVID-19 patients that presented ARDS, needed ventilator support to overcome the patient's inability to accomplish sufficient gas exchange [36]. Patients who did not survive had a persistent hypoxia beyond 72 h after ICU admission (PaO2/FiO2 < 164). Accordingly, several studies show that low PaFiO2 upon ICU admission was related to increased mortality [37, 38].

The Sequential Organ Failure Assessment (SOFA) Score is a mortality prediction score that is based on the degree of dysfunction of six organ systems [39]. Citu et al., described that SOFA is an excellent predictor of in-hospital mortality among COVID-19 patients showing that for every one-point increase in SOFA score, mortality risk increased by 1.82 [40].

Multivariable regression showed increasing odds of in-hospital death associated with Ferritin (OR = 3.31); IL-6 (OR = 9.65); PaFiO2 (OR = 4.12); NLR 24 h (OR = 7.29); and SOFA (OR = 2.29). Additionally, using principal component analysis (PCA), we were able to classify patients in two well-defined clusters. Cluster 2 composed by the selecting significant biomarkers ferritin (≥ 1225 ng/dl,) IL-6 (≥ 11 pg/ml) and NLR (≥ 22) at 24 h, PaFiO2 (≤ 164 mmHg), and SOFA (≥ 6) at 72 h, appear to have good discriminating power to group non-survivors.

Researchers worldwide are investigating the influence of COVID-19 on pathogenesis and mortality rates while taking demographic factors into account. The results of this study illustrate the significance of several biomarkers in the illness prognosis and examines how their levels might predict disease severity in a high-altitude city, providing clinicians with a tool for grouping patients.

## Conclusion

The clinical and biomolecular pattern described in this work may contribute in the early identification of severe COVID-19 patients with a high mortality risk who live in high-altitude regions, promoting earlier treatment decision-making.

## Limitations

The limited sample size and observational, monocentric design of this study restricts the significance of causal associations. It will require meta-analyses research to support the findings of this study.

### Supplementary Information

**Additional file 1:** **Supplementary Table 1.** Mechanical ventilation parameters in non-survival and survival groups. **Supplementary Figure 1.** ROC curve showing the area under the curve (AUC) and cut-off points of ventilation markers that shown significant differences in predicting COVID-19 mortality: PEEP at 48 hours, plateau pressure at 72 hours, driving pressure at 72 hours, maximum PCO2 at 24 and 72 hours. PEEP: positive end-expiratory pressure; PCO2: Partial pressure of carbon dioxide. **Supplementary Figure 2.** Box plot displaying the distribution of data and trends of Biomarkers and Hemogram characteristic in non-survival and survival groups. LDH: lactate dehydrogenase; NLR: neutrophil-to-lymphocyte ratio.

## Data Availability

The datasets used and/or analyzed during the current study are available from the corresponding author on reasonable request.

## Abbreviations

ROC: Receiver operating characteristic
COVID-19: Coronavirus disease 2019
AUC: Area under the curve
IL-6: Interleukin-6
NLR: Neutrophil-to-lymphocyte ratio
PaFiO2: Ratio of arterial oxygen partial pressure (PaO2 in mmHg) to fractional inspired oxygen
SOFA: Sequential organ failure assessment score
OR: Odds ratio
ICU: Intensive care unit
LDH: Lactate dehydrogenase
RT-PCR: Reverse transcription polymerase chain reaction
CO-RADS: COVID-19—reporting and data system
APACHE II: Acute physiology and chronic health evaluation II score
NV: Normal value
ELISA: Enzyme-linked immunosorbent assay
EDTA: Etheylenediaminetetraacetic acid
SD: Standard deviation
T2DM: Type 2 diabetes mellitus
HBP: High blood pressure
BMI: Body mass index
CI: Confidence interval
PEEP: Positive end-expiratory pressure
Ll: Lower limit
Hl: Higher limit
CATPCA: Categorical principal component analysis
ARDS: Acute distress syndrome
VT: Tidal volume
Pplat: Plateau pressure
PCO2: Partial pressure of carbon dioxide
NMBAs: Neuromuscular blocking agents
VM: Mechanical ventilation
