# Supplementary material for: Receiver operating characteristic (ROC) to determine cut-off points of clinical and biomolecular markers to discriminate mortality in severe COVID-19 living at high altitude
Source: BMC Pulm Med. 2023 Oct 18;23:393. doi: 10.1186/s12890-023-02691-2 (PMC10583315; doi:10.1186/s12890-023-02691-2)
Supplement: Supplementary file 1 — Additional file 1: Supplementary Table 1. Mechanical ventilation parameters in non-survival and survival groups. Supplementary Figure 1. ROC curve showing the area under the curve (AUC) and cut-off points of ventilation markers that shown significant differences in predicting COVID-19 mortality: PEEP at 48 hours, plateau pressure at 72 hours, driving pressure at 72 hours, maximum PCO2 at 24 and 72 hours. PEEP: positive end-expiratory pressure; PCO2: Partial pressure of carbon dioxide. Supplementary Figure 2. Box plot displaying the distribution of data and trends of Biomarkers and Hemogram characteristic in non-survival and survival groups. LDH: lactate dehydrogenase; NLR: neutrophil-to-lymphocyte ratio. [file 12890_2023_2691_MOESM1_ESM.docx]

**SUPPLEMENTARY INFORMATION**

**Supplementary Table 1.** Mechanical ventilation parameters in non-survival and survival groups.

| **Mechanical ventilation parameters** | **Total** | **Condition at Discharge** | | **p-value** |
| --- | --- | --- | --- | --- |
|  |  | **No-survival** | **Survival** |  |
| **Ventilation mode at ICU admission (n(%))^2/^** | | | | |
| Volume control ventilation | 16 (7,8) | 5 (31,25) | 11 (68,75) | 0,767 |
| Pressure control ventilation | 189 (92,2) | 66 (34,92) | 123 (65,08) |  |
| VT x Kg 24h (mean (SD)) ^3/^ | 6,89 (1,23) | 6,88 (1,24) | 6,89 (1,23) | 0,998 |
| VT x Kg 48h (mean (SD)) ^3/^ | 7,67 (6,75) | 6,95 (1,1) | 8,06 (8,28) | 0,765 |
| VT x Kg 72h (mean (SD)) ^3/^ | 7,49 (5,28) | 7,09 (1,34) | 7,71 (6,54) | 0,589 |
| PEEP 24h (mean (SD)) ^3/^ cmH20 | 9,79 (2,09) | 9,77 (2,14) | 9,8 (2,07) | 0,998 |
| PEEP 48h (mean (SD)) ^3/^ cmH20 | 8,6 (2,18) | 9,19 (2,28) | 8,29 (2,07) | **0,009*** |
| PEEP 72h (mean (SD)) ^3/^ cmH20 | 7,84 (2,07) | 8,35 (2,35) | 7,56 (1,86) | 0,072 |
| Pplat 24h (mean (SD)) ^1/^ cmH20 | 23,07 (4,01) | 23,55 (3,9) | 22,82 (4,07) | 0,217 |
| Pplat 48h (mean (SD)) ^3/^ cmH20 | 21,51 (3,71) | 22,03 (3,84) | 21,23 (3,62) | 0,103 |
| Pplat 72h (mean (SD)) ^3/^ cmH20 | 20,84 (4,41) | 22,2 (3,9) | 20,09 (4,5) | **0,002*** |
| Compliance 24h (mean (SD)) ^3/^ ml/cmH2O | 31,74 (11,99) | 31,14 (12,63) | 32,05 (11,67) | 0,583 |
| Compliance 48h (mean (SD)) ^3/^ ml/cmH2O | 32,74 (11,77) | 33,15 (12,64) | 32,53 (11,33) | 0,817 |
| Compliance 72h (mean (SD)) ^3/^ ml/cmH2O | 35,68 (9,8) | 35,56 (9,39) | 35,74 (10,07) | 0,735 |
| Driving pressure 24h (mean (SD)) ^3/^ cmH20 | 13,43 (2,97) | 13,69 (3,11) | 13,3 (2,9) | 0,378 |
| Driving pressure 48h (mean (SD)) ^3/^ cmH20 | 13,12 (2,76) | 13,43 (2,97) | 12,95 (2,65) | 0,300 |
| Driving pressure 72h (mean (SD)) ^3/^ cmH20 | 13,05 (2,86) | 13,67 (3,11) | 12,71 (2,66) | **0,024*** |
| Mechanical Power 24h (mean (SD)) ^3/^ j/min | 15,67 (4,19) | 15,85 (4,14) | 15,57 (4,23) | 0,645 |
| Mechanical Power 48h (mean (SD)) ^3/^ j/min | 14,94 (3,91) | 14,72 (3,69) | 15,05 (4,03) | 0,755 |
| Max. PCO_2_ 24h (mean (SD)) ^3/^ mmHg | 44,82 (12,99) | 46,95 (13,34) | 43,69 (12,7) | **0,041*** |
| Max. PCO_2_ 8h (mean (SD)) ^3/^ mmHg | 44,66 (11,28) | 47,1 (12,77) | 43,44 (10,29) | 0,074 |
| Max. PCO_2_ 72h (mean (SD)) ^3/^ mmHg | 43,35 (11,99) | 47,49 (15,08) | 41,26 (9,48) | **0,002*** |
| PaFiO_2_ 24h (mean (SD)) ^3/^ mmHg | 146,42 (49,17) | 131,89 (45,05) | 154,12 (49,68) | **0,010*** |
| PaFiO_2_ 48h (mean (SD,)) ^3/^ mmHg | 168,37 (43,32) | 154,79 (34,79) | 175,16 (45,63) | **0,005*** |
| PaFiO_2_ 72h (mean (SD)) ^1/^ mmHg | 174,04 (49,06) | 148,12 (38,96) | 187,1 (48,54) | **0,000*** |
| Prone positioning ventilation (n (%)) ^2/^ | 126 (61,46) | 48 (38,1) | 78 (61,9) | 0,188 |
| Days of prone positioning (mean (SD)) ^3/^ | 1,85 (1,4) | 1,98 (1,42) | 1,78 (1,39) | 0,489 |
| Use of NMBAs (n (%)) ^2/^ | 128 (63,37) | 47 (36,72) | 81 (63,28) | 0,313 |
| Days with NMBAs (mean (SD)) ^3/^ | 1,79 (1,42) | 1,98 (1,51) | 1,69 (1,37) | 0,323 |
| Days with VM (mean (SD)) ^3/^ | 8,64 (6,97) | 10,39 (7,18) | 7,7 (6,69) | **0,002*** |
| **Extubation (n (%)) ^2/^** | | | | |
| Failure | 45 (28,13) | 27 (60) | 18 (40) | **0,000**** |
| Success | 115 (71,88) | 2 (1,74) | 113 (98,26) |  |

ICU: Intensive care unit; SD: Standard deviation; VT: tidal volume; PEEP: positive end-expiratory pressure; Pplat: plateau pressure; PCO2: Partial pressure of carbon dioxide; PaFiO2: ratio of arterial oxygen partial pressure (PaO2 in mmHg) to fractional inspired oxygen; NMBAs: neuromuscular blocking agents; * Significant differences, based on 1/ T-test y 3/ Mann Whitney U test; ** Significant differences in the no-survival condition, based on Chi-square test or exact test of Fisher 2.


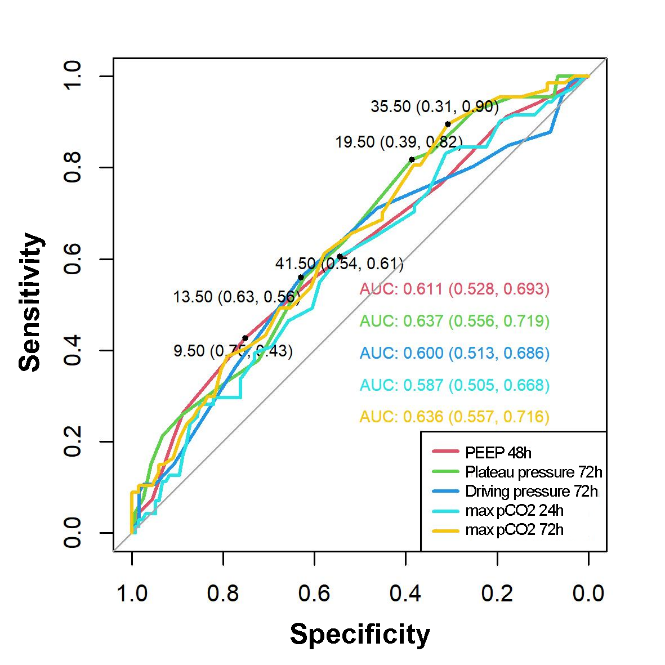


**Supplementary Figure 1:** ROC curve showing the area under the curve (AUC) and cut-off points of ventilation markers that shown significant differences in predicting COVID-19 mortality: PEEP at 48 hours, plateau pressure at 72 hours, driving pressure at 72 hours, maximum PCO2 at 24 and 72 hours. PEEP: positive end-expiratory pressure; PCO2: Partial pressure of carbon dioxide.


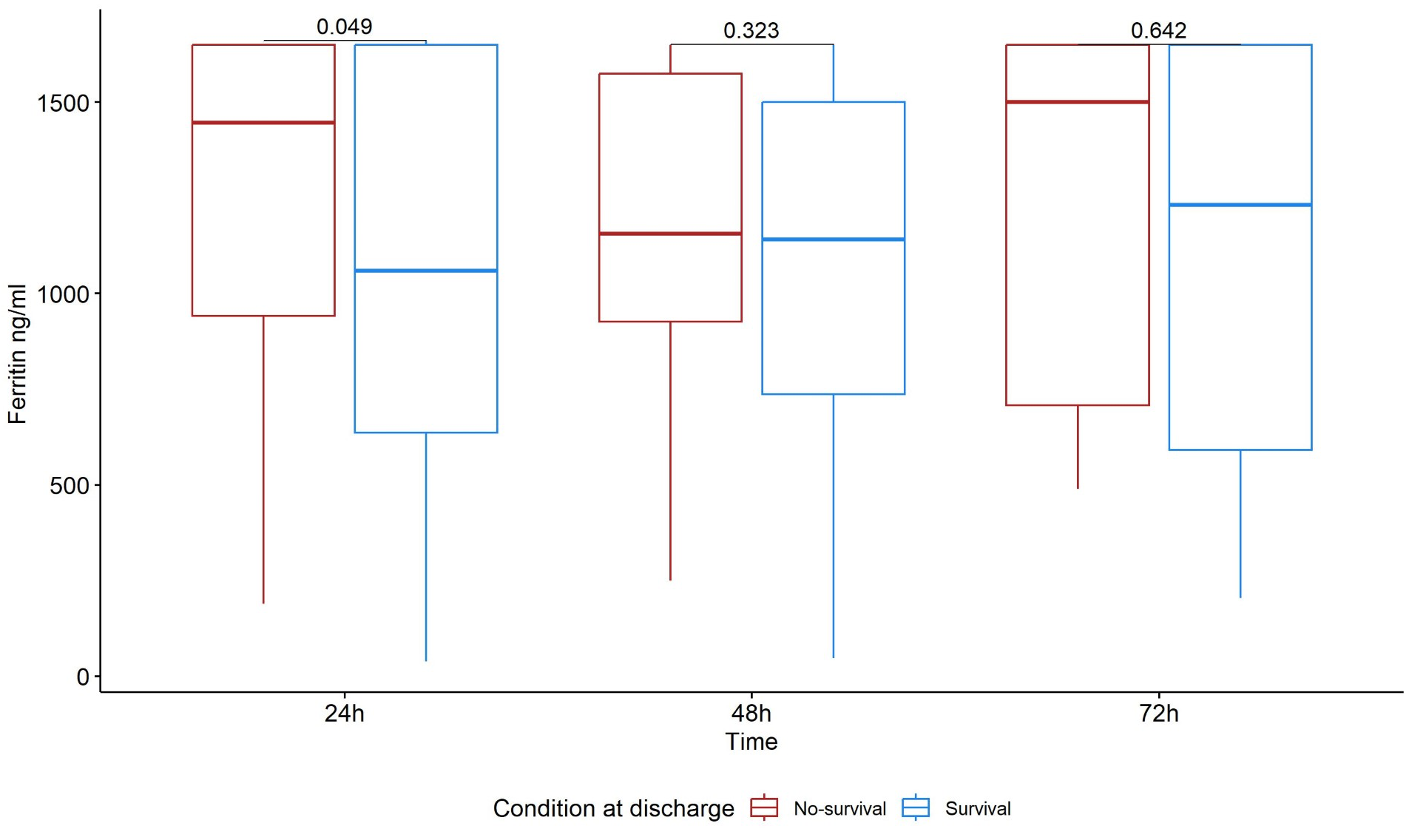

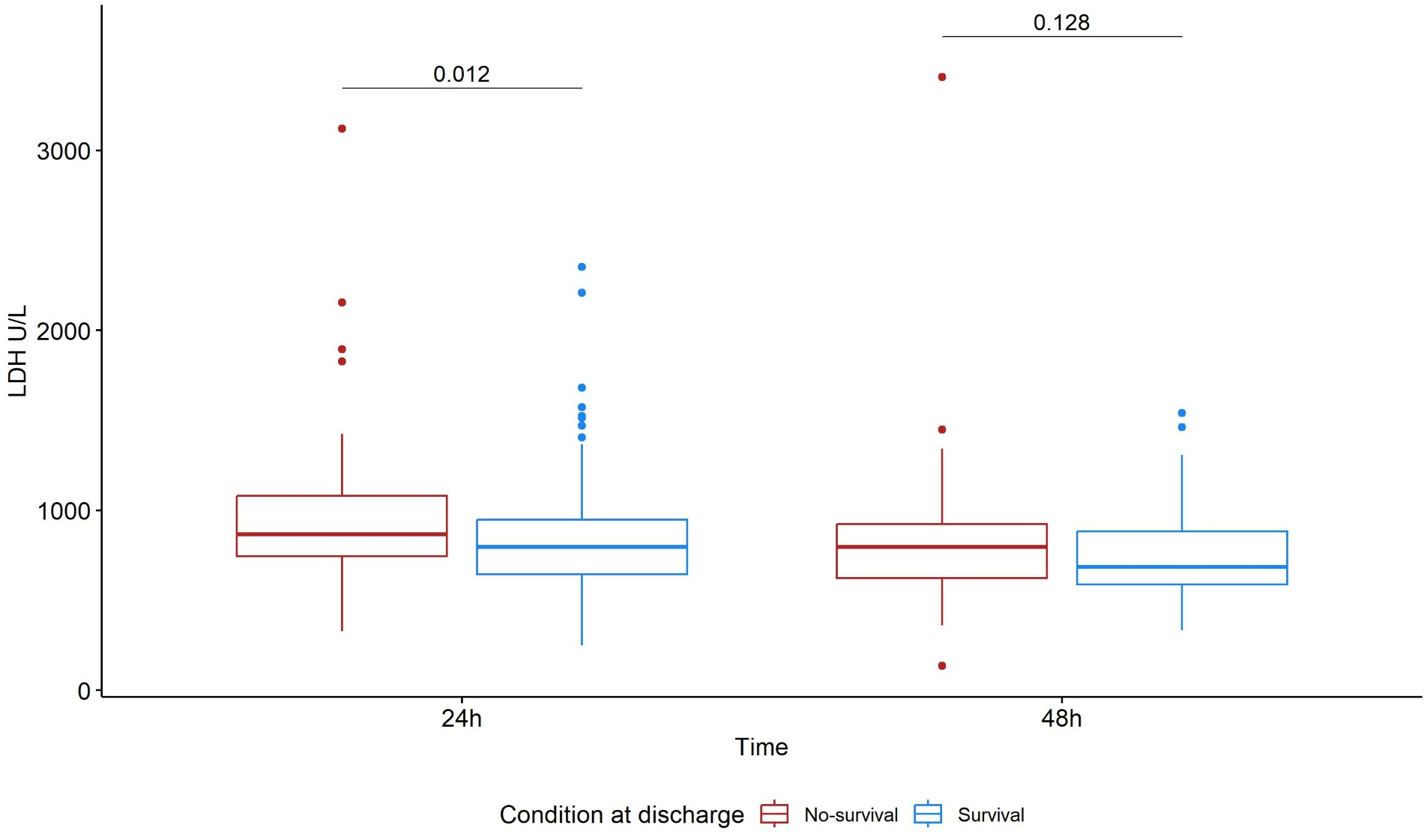

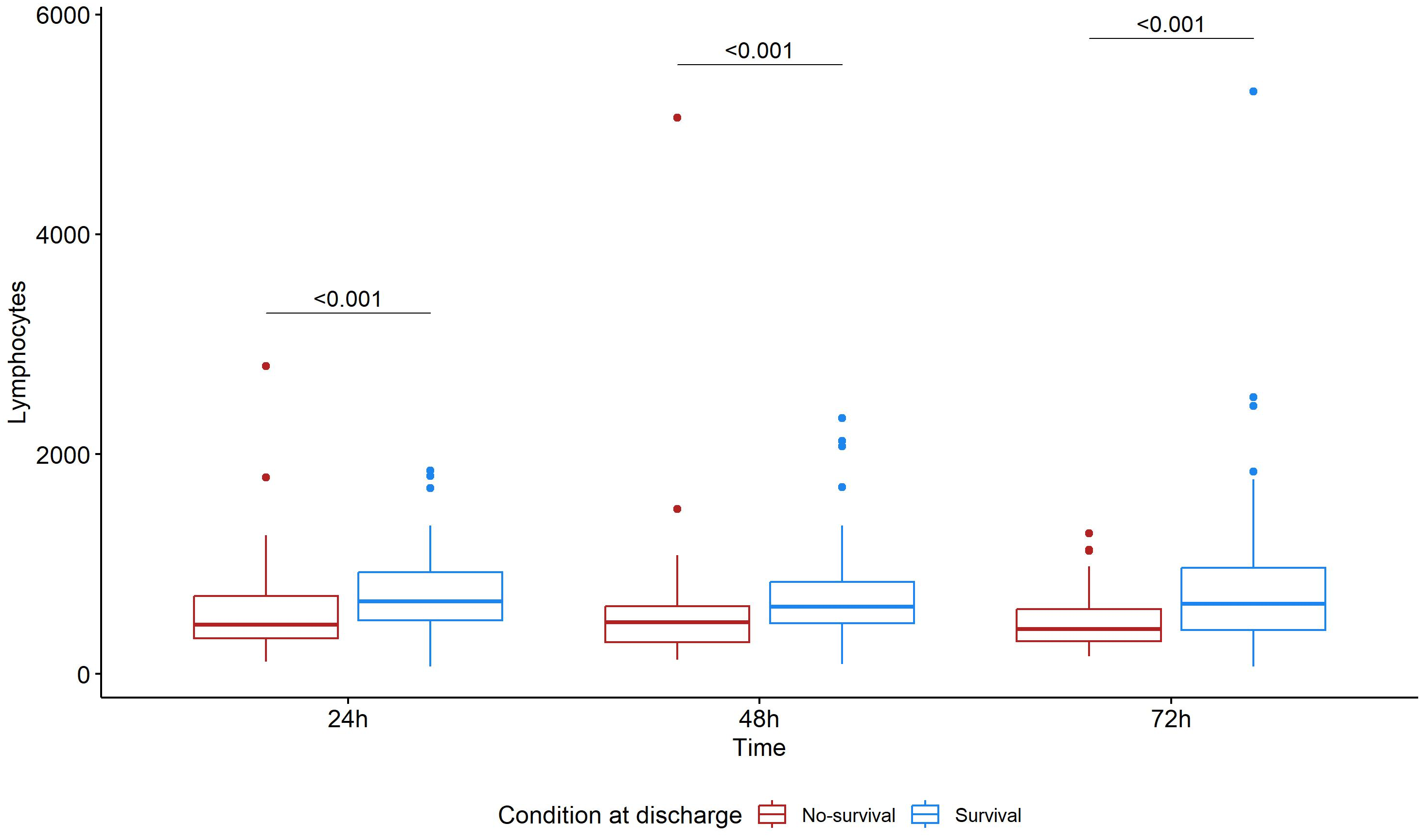

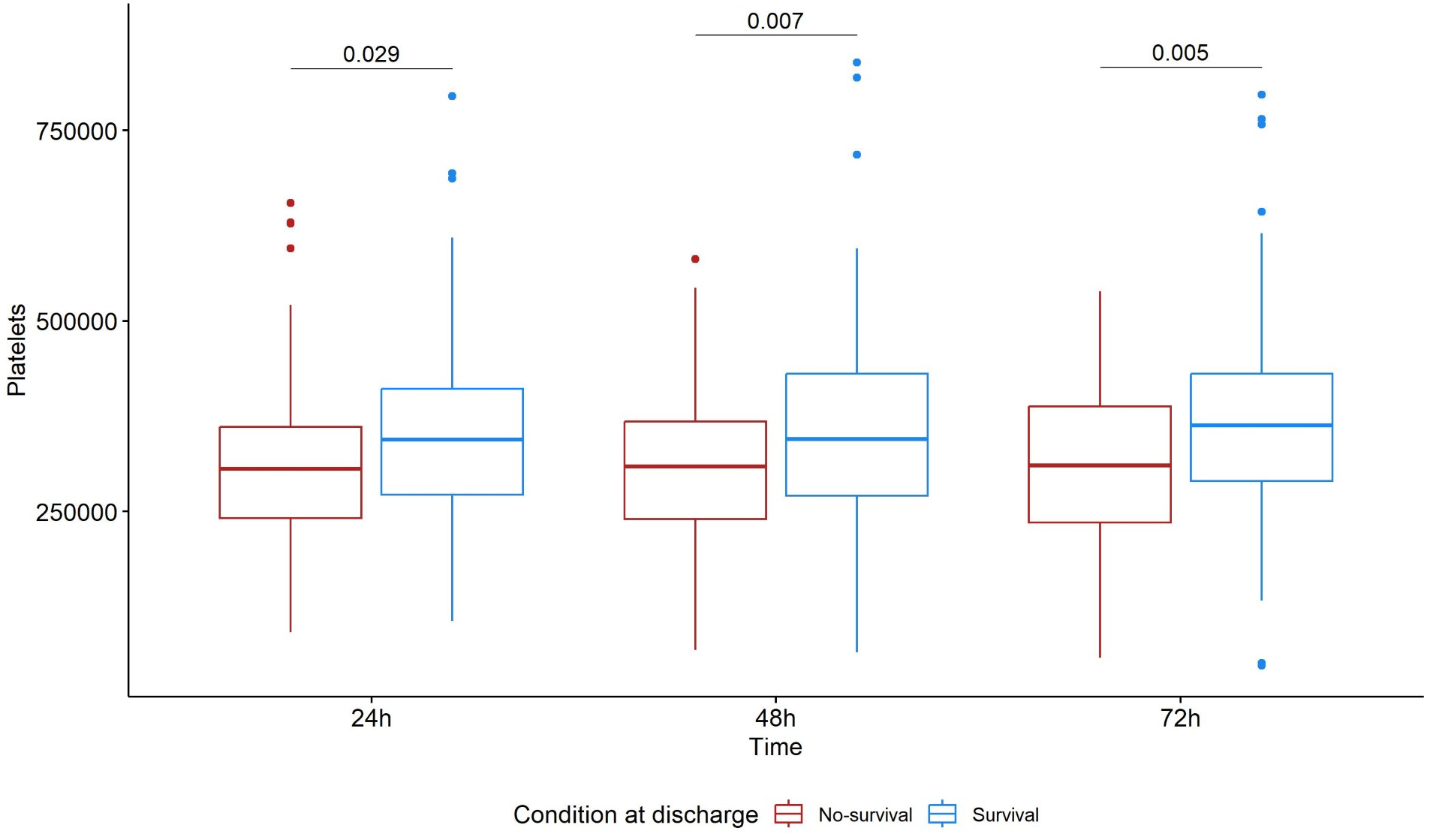

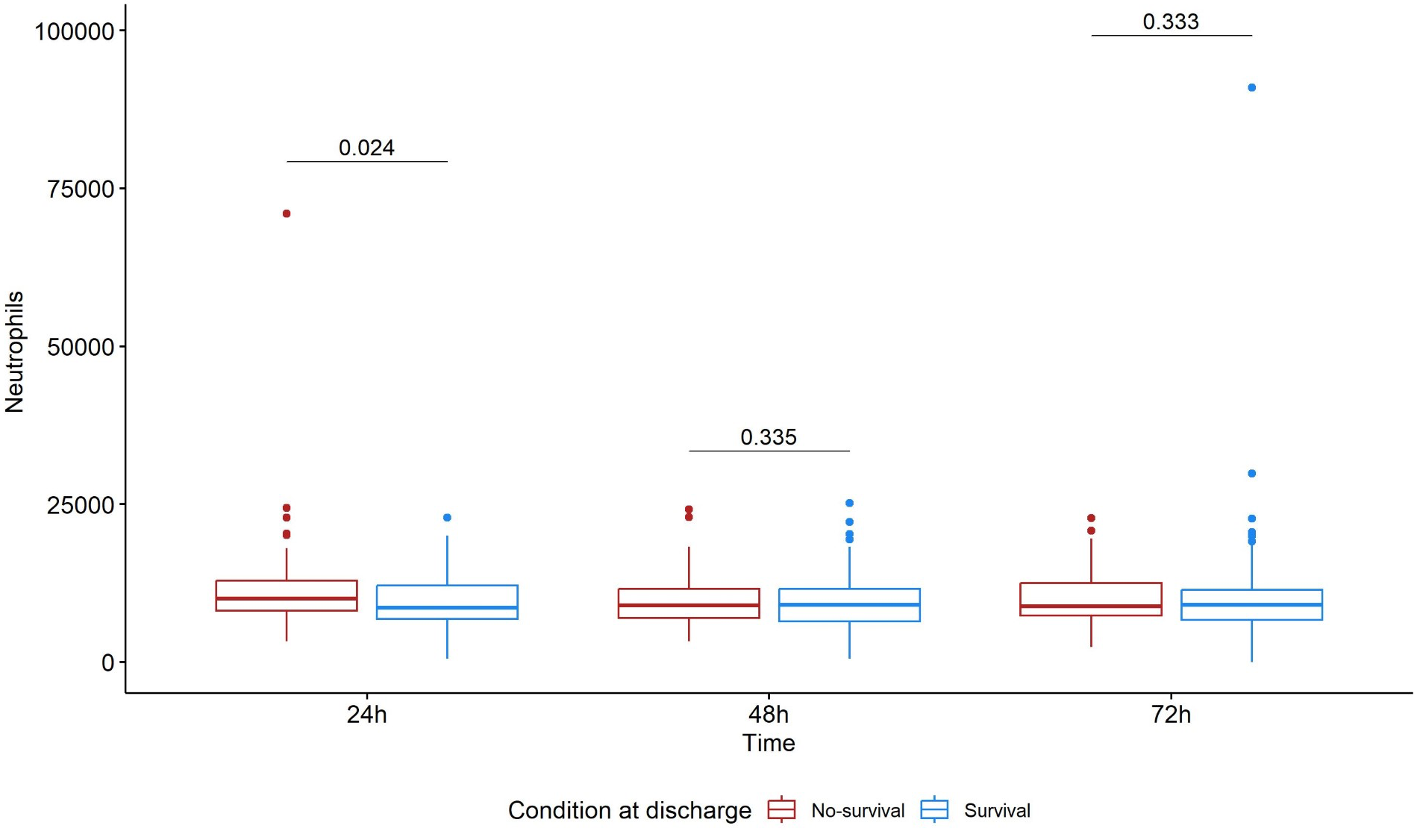

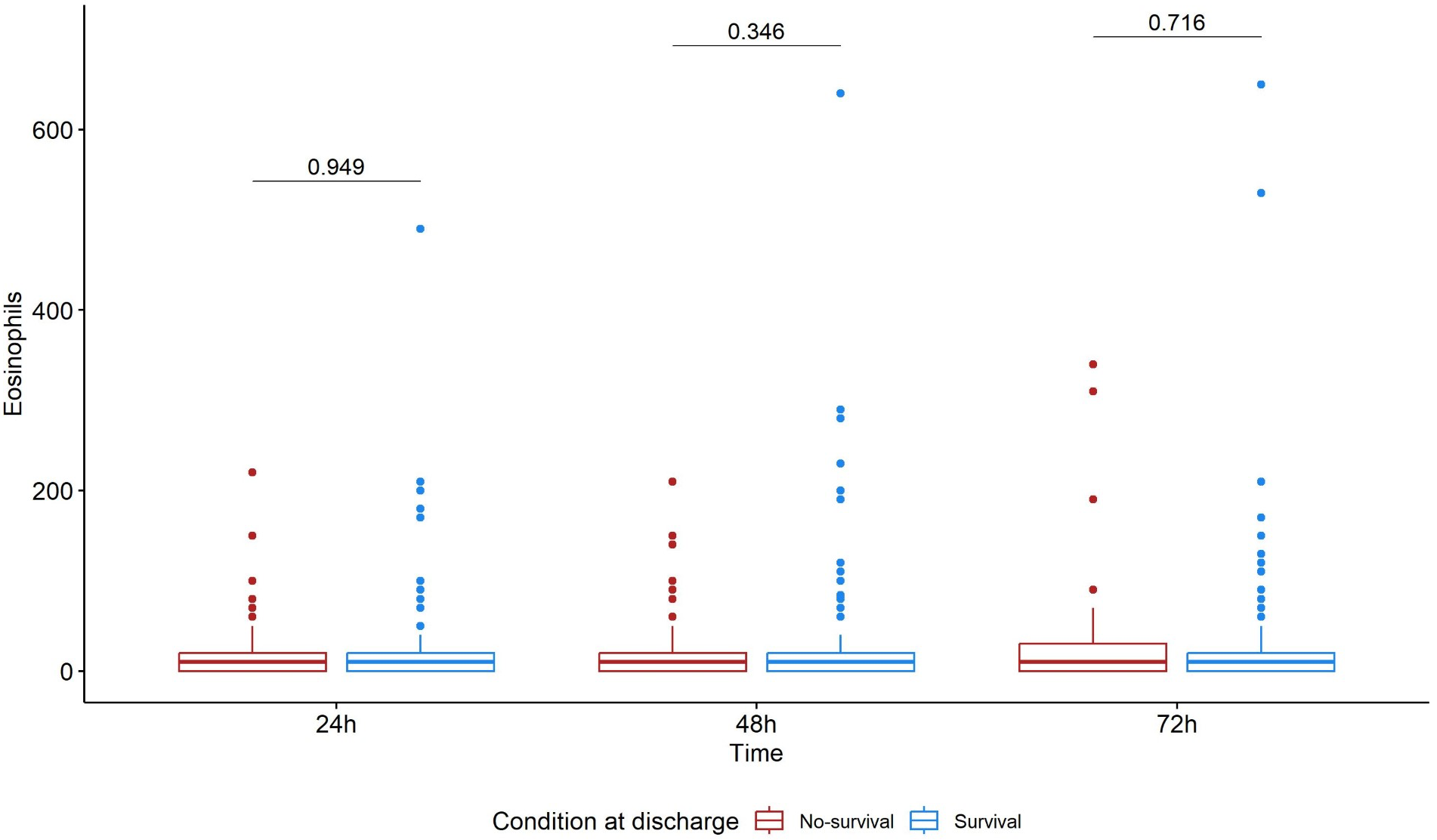

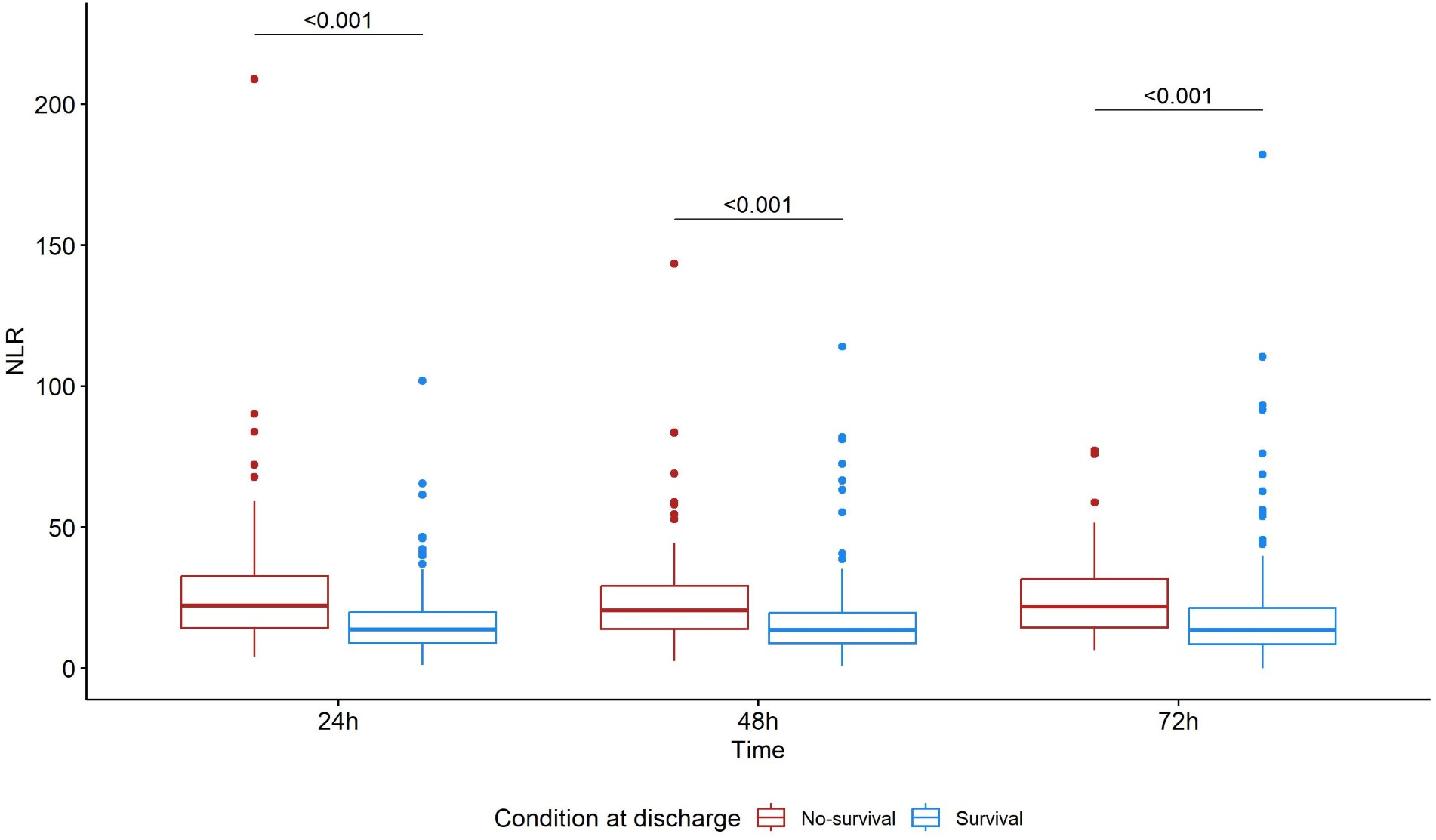


**Supplementary Figure 2:** Box plot displaying the distribution of data and trends of Biomarkers and Hemogram characteristic in non-survival and survival groups. LDH: lactate dehydrogenase; NLR: neutrophil-to-lymphocyte ratio
